# Screening State of Play: The Biosecurity Practices of Synthetic DNA Providers

**DOI:** 10.1089/apb.2023.0027

**Published:** 2024-06-20

**Authors:** Arianne Kane, Michael T. Parker

**Affiliations:** ^1^ Department of Biology, Georgetown University, Washington, DC, USA.; ^2^ College of Arts & Sciences, Georgetown University, Washington, DC, USA.

**Keywords:** synthetic DNA, regulation, screening, biosecurity, synthetic biology

## Abstract

**Introduction:**

Synthetic DNA technology is rapidly emerging as a key driver of innovation in the fields of medicine, biotechnology, and more. But it also poses significant risk, particularly in lowering barriers to the production of dangerous pathogens and toxins. At present, oversight of this technology is voluntarily coordinated among synthetic DNA providers and stakeholders, and detailed understanding of security processes, infrastructures, and insights from these providers is imperative to understand how to best mitigate the inherent risks of this technology.

**Objectives:**

In this study, we aimed to determine the trends, outliers, strengths, and gaps in current DNA provider security practices through a broad survey of the gene synthesis field.

**Methods:**

We interviewed synthetic DNA providers and stakeholders about their customer and sequence screening procedures. Respondents were divided into groups based on membership in the International Gene Synthesis Consortium, nationality, whether they were a new or established company, and whether they synthesize de novo DNA or not. We then performed meta-analysis and intergroup analysis to elucidate larger trends and points of variance.

**Results:**

In total, we interviewed 18 companies. We found that synthetic DNA providers and stakeholders tend to operate under a “zero-trust model” for screenings and utilize common governmental and private resources to navigate international import/export policies. Major variabilities were identified in the sensitivity of screening, monitoring and evaluation practices, screening pipelines, and approaches to synthetic oligonucleotide screening. In addition, we identified a significant vulnerability of lacking awareness among providers of formal law enforcement reporting procedures.

**Conclusion:**

Collectively, we observed significant heterogeneity in security practice throughout the field, reflective of the current lack of codified oversight for DNA synthesis. The results presented in this study provide insight into the specifics, strengths, and shortcomings of current DNA provider security practices, and are important considerations for the biosecurity community in ongoing deliberations of if, when, and how to approach oversight of synthetic DNA technology.

## Introduction

In the past quarter century, advances in DNA synthesis technologies have enabled the establishment of a privatized nucleotide sequence synthesis industry. Increased technical capabilities, paired with declining costs, now allow these providers to produce accurate de novo sequences quickly and at a reasonable cost.^1^ Easy access to established sequences, paired with generation of completely novel sequences, is enabling new avenues of research that were, until recently, difficult to impossible.

Consideration for establishment of safeguards has been a point of discussion for many years in the DNA synthesis field.^2,3^ Indeed, the biosecurity community has expressed concern^4^ that synthetic DNA technologies have lowered barriers that previously stood in the way of acquisition of dangerous genetic material by both good and bad actors, thus increasing the chances of accidental or intentional biothreat incidents.

The U.S. Department of Health and Human Services (DHHS) *Screening Framework Guidance for Providers of Synthetic Double-Stranded DNA*^4^ has been the leading document for security considerations in DNA sequence and customer screening since 2010.^5^ But the Guidance is nonbinding, and to date, synthetic DNA providers and stakeholders have self-governed through the International Gene Synthesis Consortium (IGSC).^6^ Founded in 2009, the IGSC is a voluntary coalition of synthetic DNA providers and stakeholders who operate under a Harmonized Screening Protocol.^7^ The Protocol provides consensus screening procedures to verify the legitimacy of purchasers and the safety of potential sequences, hoping to identify and deny bad actors or dangerous orders before synthesis.

Despite this common workflow, the lack of regulation for sequence and customer screening has naturally led to inconsistencies throughout the field, some of which might be exploitable by bad actors. Indeed, many stakeholders have expressed the opinion that there is a need for a universal screening mechanism and improved governance of the synthetic DNA field.^6^ In response, DHHS has been working with the biosecurity community to better understand the technical threat landscape for DNA synthesis, and recently published updated Guidance for providers.^8–10^ These deliberations influenced the President's *Executive Order 14110*, which has a section that compels relevant U.S. Government agencies to create a framework for sequence and customer screening by April 27, 2024.^11^

To better inform these ongoing discussions of if, when, and how to regulate access to synthetic DNA technology, it is imperative to understand the practices currently in use by providers. In this study, we report our efforts to clarify the current landscape of screening procedures through interviews with individual synthetic DNA providers and stakeholders. From these discussions, we have identified trends, outliers, strengths, and gaps in screening protocols.

Importantly, we have noted striking variabilities between providers in regard to treatment of synthetic oligonucleotides, monitoring and evaluation of sequence screening procedures, and sensitivity of sequence screens, as well as a concerning lack of protocols for law enforcement reporting. These findings detail important context directly from the entities that would be most impacted by regulatory action, and provide useful and concrete considerations for governments around the world when deciding the future of DNA synthesis guidance and governance.

## Methods

Synthetic DNA providers and stakeholders, encompassing made-to-order DNA synthesis companies (including benchtop synthesis organizations), providers of cloned DNA, security screening providers, and stakeholders involved in policy and strategy, were targeted for interviews. Potential contacts were identified from the IGSC's website,^12^ Google searches for keyphrases such as “DNA synthesis companies,” and from articles related to synthetic gene technology.^13,14^

When possible, individuals most relevant to sequence screening were identified from public staffing lists on company websites, but when those were not available, general email addresses and online contact forms were utilized. In some cases, when emailing was not effective, company representatives were contacted through phone or through networking outreach on LinkedIn. Inquiries for interviews were sent to 56 providers and stakeholders in total.

A literature review on gene sequence screening was conducted to determine key questions related to gene synthesis, biosecurity, and screening procedures. Then, interview questions were developed (see Supplementary Data for complete list) that addressed two overarching questions:

1. How does your organization approach screening orders and customers, philosophically and technically?
2. How does your organization approach international ordering of synthetic sequences, if at all, from export control, handling, and etc. standpoints?

Interviews were conducted from September to November of 2023, notably, after a request for information from DHHS on proposed screening Guidance,^8^ but before providers would have had time to react to the eventual Guidance published in late October 2023.^10^ Interviewees were informed of the goals of the study before their interview took place. All answers were de-identified during drafting of this article and respondents were given the opportunity to review the draft article before publication so that they could ensure privacy for themselves and their company.

Interviews were opted in and conducted in three modalities: interviews with recordings, interviews without recordings, and interviews with written responses. Interviews with recordings were conducted over Zoom, with the meeting and automatically generated transcripts recorded to the Cloud. Auto-generated transcripts were reviewed and corrected to ensure accuracy within 2 days of the interview.

For interviewees who did not want their interviews recorded, typed notes were taken instead, and for interviewees unable or unwilling to sit for formal interviews, the full written list of interview questions was provided, and answers were returned in written form. Follow-up questions were asked over email in response to ambiguous language or insufficient detail. Requests for the destruction of interview videos, transcripts, and notes after the publication of the study were fielded and fulfilled.

## Results

### Overview of Respondents and Responses

Of the 56 synthetic DNA providers and stakeholders contacted, 13 did not respond, 15 declined an interview, and 4 declined an interview but provided some information over the phone or through email. Six providers and stakeholders started the process of scheduling an interview or providing written responses, but stopped responding to inquiries during the process. Ultimately, 17 providers and stakeholders sat for interviews and 1 provider submitted written responses. One interviewee represented both their company and the IGSC in their interview. Respondents ranged in size from laboratories of <10 people to multinational corporations with >50 international locations and billions of dollars of yearly revenue.

Of the 18 organizations that provided input, 14 were IGSC members and one was the IGSC itself, representing 83.3% of the respondent pool. Six providers and stakeholders were international, meaning that their global headquarters are based in a country that is not the United States. In the case of multinational corporations, providers whose gene synthesis services were based outside of the United States but whose global headquarters were domestic were considered international.

**Figure 1.**
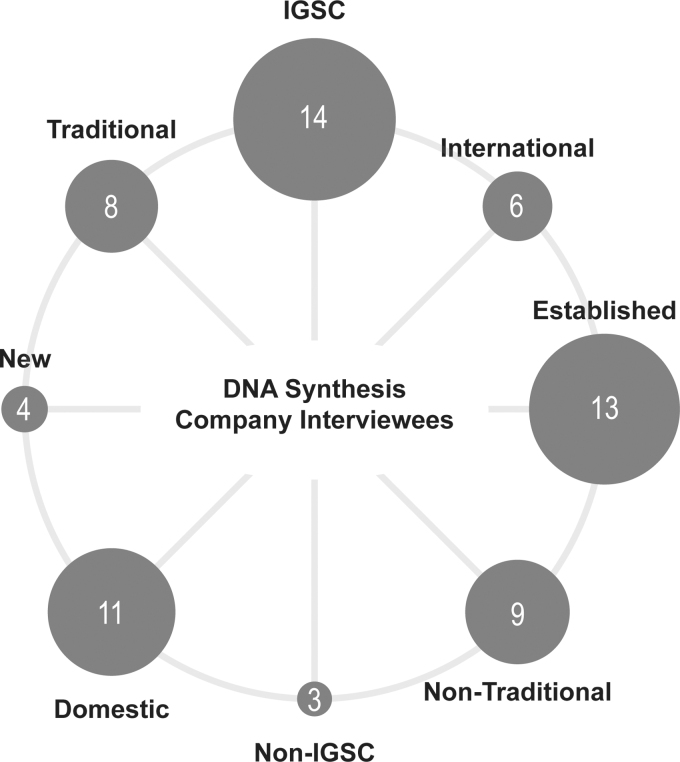
Classification of interviewed organizations. Respondents were organized into eight categories for this analysis. Note that each company was assigned to exactly four of these categories, as it is a set of four either/or classifications. These figures do not include the IGSC itself, the 18th respondent. IGSC, International Gene Synthesis Consortium.

Four providers and stakeholders were designated as “new,” signifying that their synthesis business and screening procedures are still in the process of being created or finalized. Nine providers and stakeholders were nontraditional, in that they do not do de novo gene synthesis, but work in directly related capacities. Examples include sequence screening companies, oligonucleotide production companies, providers of stock DNA such as plasmids, and vendors of benchtop synthesis devices (Figure 1). These classifications do not include the IGSC itself, who was the 18th respondent.

Across all interviews, trends emerged in a few key areas: technical aspects of screening, cost considerations, security measures, and synthesis hardware. Summaries of the most notable takeaways on these topics are provided in Table 1. In the following sections, we will provide detailed description of individual and collective provider and stakeholder responses for each of these topics.

**Table 1.** Notable findings across interviewees

Reference sequence of concern databases are sometimes built in-house, but can be contracted out. Even with IBSB, no two companies seem to do the act of screening exactly the same.
Approaches to synthetic oligonucleotide screening are varied, although more often than not synthetic oligonucleotides are not screened at all.
Screening practices, including monitoring and evaluation, are variable in their implementation and their robustness.
Law enforcement reporting procedures are not often utilized, and alarmingly unclear to some companies. This is perhaps the most pressing vulnerability we uncovered.
Import/export flags are a large proportion of screening flags. Continued, and perhaps expanded, support for navigation of regulations for import/export is prudent.
Most companies have a “zero-trust” model for sequences and customers, although some sequence whitelisting occurs.

### Technical Trends in Screening

Most commonly, providers screen potential purchasers of synthetic DNA by verifying their identity and association with a trusted organization who have a legitimate use for synthetic DNA. Requested sequences are also computationally compared with pathogenic sequences of concern (SOCs). Primary customer and sequence screening can be conducted in parallel, but some providers will screen customers before allowing an order to be submitted. Any sequences that are a best match to an SOC are flagged for human review.

**Figure 2.**
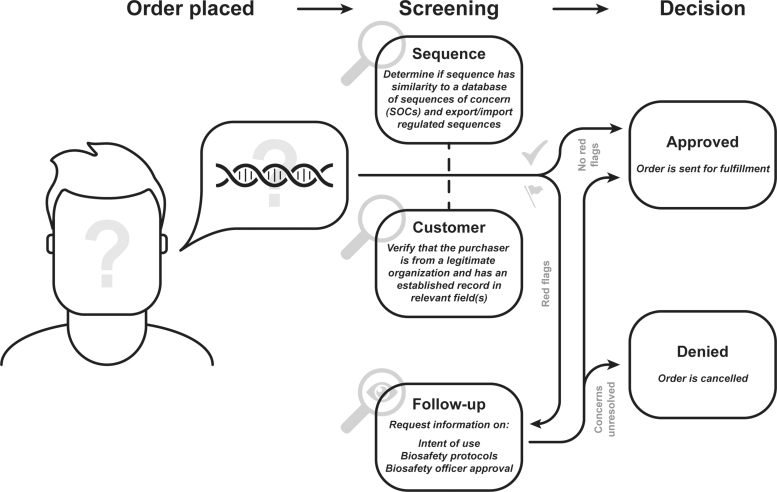
Consensus workflow of DNA sequence and customer screening. This general framework is a consensus drawn from 18 interviews conducted with synthetic DNA providers and stakeholders.

In the event of a flagged order or customer, providers conduct a follow-up with the customer either to obtain proper export licenses or verify that the purchaser has legitimate business with the sequence and the proper biosafety protocols to handle it. The order then is either allowed or terminated based on the results of the follow-up screening (Figure 2).

Across the providers interviewed, a core set of guidelines have informed the development of screening procedures (Table 2). In general, synthetic DNA providers utilized some combination of the DHHS 2010 Guidance,^15^ the U.S. Commerce Control List,^16^ the U.S. Select Agents and Toxins List,^17^ the European Union's Control List of Dual-Use items,^18^ and the Australia Group Common Control List.^19^

**Table 2.** Common guidelines informing screening protocols

Australia Group Biological Agent List
EU Dual-Use Items List
DHHS Screening Framework Guidance for Providers of Synthetic Double-Stranded DNA
IGSC Harmonized Screening Protocol
U.S. Commerce Control List
U.S. Select Agents and Toxins List

DHHS, Department of Health and Human Services; IGSC, International Gene Synthesis Consortium.

Some frameworks, such as the Australia Group List, EU Dual-Use List, and DHHS Guidance, are broad enough that they encompass the regulations of multiple countries at once. For example, one benchtop synthesis company noted that their screening procedures for United States and EU markets were highly similar due to both regions falling under the Australia Group guidelines. Another international synthetic DNA provider claimed that by following the Australia Group List, EU Dual-Use List, and DHHS Guidelines, they were largely able to universalize their screening across the United States and Europe.

Of course, IGSC member organizations designed their screening procedures for compliance with the most recent IGSC Harmonized Screening Protocol in addition to the regulations described earlier, which largely concern how to designate SOCs and considerations for export control rather than delving into the screening procedure itself. The IGSC Protocol seems to be especially formative for the new providers interviewed, all of which were already members of the IGSC despite not yet finalizing their screening procedures.

SOC lists utilized in screening are acquired similarly by the providers and stakeholders interviewed, although there is a divide between those that build their own systems and those that pay an external screening service provider. Providers that have internal pipelines to screen for SOCs (9 of the 13 established providers, excluding the IGSC and the 1 respondent that is a sequence screening company) generally drew upon lists of SOCs designated by the U.S. Select Agents and Toxins List, U.S. Commerce Control List, and Australia Group List.

IGSC organizations also have access to the consortium's own Regulated Pathogen Database (RPD), which draws from the Australia Group, U.S. Select Agents and Toxins List, EU guidelines, and other common control frameworks. In general, these providers screen potential orders against SOCs by utilizing a sequence alignment tool, of which the National Center for Biotechnology's Basic Local Alignment Search Tool (BLAST) was most popular (cited by seven respondents).^20^

The majority of providers who use BLAST have incorporated it into an automated screening algorithm, but one respondent reported conducting BLASTs of orders manually as they first initiated their screening protocols. In contrast, three established providers and two new providers contract with sequence screening providers, most commonly those developed by Aclid and Battelle. The exact nature of these screening methods is proprietary, but respondents reported using similar frameworks to those of synthetic DNA providers who build their own databases in-house.

There remains major variability in the implementation of common protocols across member organizations. At the moment, the Harmonized Screening Protocol dictates screening genetic sequences of 200 nucleotides (nt) or more (note that the Protocol refers to base pairs, but nt is effectively analogous for screening purposes while being inclusive of double- and single-stranded sequence lengths). At the time that most interviews were conducted, DHHS was about to finalize updated Guidance that recommended screening down to 50 nt.

Thus, only six IGSC organizations actually reported 200 nt as their lower bound for screening; the rest reported either screening all incoming orders regardless of length, or having lower bounds from 20 to 60 nt. New organizations had more stringent procedures on average, with three reporting a screening lower bound from 20 to 50 nt and one claiming that they will screen everything. This could reflect a higher degree of cautiousness among newer providers, or perhaps just that the public discourse around this issue has clearly trended toward screening shorter sequences.

It is also possible that because newer organizations receive less orders, they have more bandwidth to screen smaller sequences. The now-finalized DHHS Guidance gives synthesis providers 3 years to make their lower bound for screening 50 nt (or less), so this current distribution of screening bounds is likely to change in the near future.

The treatment of single strands of DNA or RNA, known as oligonucleotides, by sequence screening procedures is a major area of diversity of approach. Of the seven organizations who produce oligonucleotides, all but one either do not screen them at all (three respondents) or do not screen them if their lengths fall below the provider's standard lower bounds for screening (four respondents). In the second case, the majority of oligonucleotide sequences produced would not be screened, but longer single-stranded nucleotide sequences that fall above the regular screening threshold would.

One provider reduces their lower bound for screening to 30 nt when screening oligonucleotides. Another new provider reported that they plan to screen oligonucleotides for SOC sequence motifs, but have yet to concretize their procedure. The most innovative approach to oligonucleotide screening was proposed by a sequence screening provider, who currently screens every order of 50 nt or longer, but surmised that if in the future they receive high quantities of oligonucleotide orders that may be for building genes, it is possible to assess which are thermodynamically capable of assembly into a larger sequence. This analysis would reduce the pool of oligonucleotides that require further screening.

### Cost Consideration Trends in Screening

The cost of implementing screening procedures is also treated differently across IGSC providers. Three providers with internal screening databases mentioned that they did not believe their procedures to be too costly, but the rest of the established IGSC providers interviewed said that there was a noticeable cost. A few pointed out that the largest cost was in time, as the review of flagged sequences by humans slows down the screening process. However, many providers who acknowledged the cost of screening also noted that this cost was reasonable in comparison with the cost of producing a dangerous sequence.

Others simply considered the marked cost of screening to be part of the standard operating costs of running a synthetic DNA company. Furthermore, there was no significant difference in attitudes toward cost between providers screening to 200 nt versus 50 nt. This is likely because those who screen to 50 nt made the choice to do so before DHHS formalized their recommendations for every provider to screen to 50 nt and thus were already willing to take on the elevated time cost associated, self-selecting as a group to be tolerant of the cost burden.

Strategies to mitigate the cost of screening are varied. Seven entities pass at least some of the cost of screening or navigating import/export procedures on to the customer. The utilization of free alignment software such as BLAST can also reduce the price of screening for those with internal screening databases. Automation (reported by four respondents) also reduces the cost of internal pipelines, as this reduces the time and financial cost of human review.

The annotation and whitelisting of sequences that have previously undergone human review and approval further automates the process and reduces the time cost, as mentioned by one company. Contrarily, three interviewees claimed that their internal screening pipeline reduces the cost of screening, whereas two others said that contracting with an external screening provider is more cost-effective.

To further mitigate the time and monetary cost of screening, 10 providers have standardized their screening procedures across both international and domestic purchases. In these cases, many used SOC databases based on broad lists of SOCs (i.e., the Australia Group list). The workflow of screening remains the same, with potential orders being run through standard screening algorithms and the same data being collected on customers.

### Security Trends in Screening

Monitoring and evaluation (M&E) of screening procedures takes different forms between providers, when it exists at all. Seven providers conduct some sort of evaluation of their procedures on a random or regular basis, whereas the rest do not, sometimes claiming that the automation of their procedures makes M&E unnecessary (although in the case of new companies, there are not yet established procedures to review). In three cases of automation, the interviewees claim that human review of flagged sequences allows them to identify any errors in their system as they occur. Or, they state that their automated screening system will not allow an order to proceed if it has not been checked against their SOC database.

Those who do conduct regular or random M&E most commonly run “test sets” that contain predefined quantities of SOCs. They can then evaluate how accurately their system identifies these sequences and make updates as needed. System red-teaming was also common, whereby a trusted entity attempts to circumvent sequence screening procedures to identify loopholes.^21^ One international provider based in Germany noted that they also receive both regular and unannounced audits from the German authorities.

Very few providers interviewed had defined procedures for the reporting of suspicious orders to law enforcement. Seven respondents at least knew that they could report concerns to the U.S. Federal Bureau of Investigation (FBI), but only four of those said with certainty that company protocol calls for such reporting. One domestic provider specifically cited their regional FBI Weapons of Mass Destruction Coordinator. Overall, five of the six international respondents did not have defined points of contact for operations outside of the United States.

The IGSC has their own internal reporting mechanism for entities to share information on suspicious orders and persons, but there is no obligation to use this system. In fact, only one IGSC respondent mentioned it unprompted during their interviews. None of the non-IGSC organizations reported having a formal suspicious order reporting procedure. Many respondents defended their lack of formal procedures by noting that they have never encountered a circumstance where they would need to report an order to the authorities, and this rarity is corroborated by providers that have reported a suspicious order in the past, having had to do so only once or twice.

Major variations in international screening procedures revolve around import–export laws and licensing. If a sequence is flagged by a screening algorithm as requiring a license for its import or export, providers have to make sure that they and their customers go through a governmental procedure to obtain proper permits to ship the sequence. Four providers will collaborate with major shipping companies such as FedEx to ensure proper permitting and/or work with in-country distributors to verify the legitimacy of international purchasers.

Only two IGSC organizations surveyed expressed willingness to fully share their screening protocols. The IGSC's Harmonized Screening Protocol is publicly available, revealing the procedures of some IGSC organizations to an extent, but without disclosing any of the variations in implementation undertaken by individual providers. Otherwise, transparency varies on a spectrum from only disclosing procedures to customers or stakeholders to not disclosing procedures at all.

In addition, no provider has a list of purchasers who are automatically considered legitimate to procure an SOC. In the interest of reducing the time spent on human review, one provider stated that they will not conduct follow-up screening with a repeat purchaser of a specific SOC if they have previously cleared follow-up screening for the sequence. If a purchaser attempts to buy an SOC distinct from past SOCs they have been cleared to purchase, further follow-up screening will be conducted. No institution or individual has *carte blanche* ability to order any SOC they want, even if they have been cleared to purchase SOCs in the past, at any of the providers interviewed.

Current guidance around the maintenance of order and customer data varies, with DHHS guidelines calling for the storage of data for at least 3 years, and, when not a burden, up to 8 years.^10^ Meanwhile, IGSC protocols state that all records should be kept for 8 years.^7^ In practice, all but one respondent maintain their data for at least 8 years. In addition, 11 respondents keep their data for 10 years or more, or report that they have never purged this data.

Two nontraditional providers that sell previously generated DNA sequences (aka, those that are not made-to-order) utilize material transfer agreements (MTAs) as a biosecurity measure. These legal documents are typically used to either prohibit the resale of a synthetic sequence provided by the company, or mandate that any third-party purchaser undergo the same customer screening as the distributor.

In one case, an interviewee claimed that the usage of an MTA legally obligated the safe handling of the DNA by their receiving customer, removing some of the need for M&E at the distributor end. This respondent noted, however, that MTAs take time to write, increase the time cost of screening, and may not be feasible for providers whose business model relies on fast turnaround time.

### Special Considerations for Benchtop Synthesis Devices

The customer and synthesis screening processes for providers of benchtop DNA synthesis devices is an emerging issue, and the current landscape has been described previously.^22^ Three of the IGSC providers and stakeholders interviewed sell benchtop synthesis devices, and their interviews provided some insight that is germane to this article. Two of the benchtop providers are new companies and indicated that they are considering using a cloud-based approach to screen sequences, where their benchtop devices send all input sequences back to the manufacturer for in-house screening before proceeding with synthesis.

These providers also claimed to be working within a “closed-loop system,” where reagents for synthesis can only be provided by the manufacturer of the synthesis devices. Being able to monitor the purchase of reagents gives benchtop companies further understanding of and control over the usage of their products. It is unclear whether such a “closed-loop system” approach would provide security against SOC synthesis, but certainly there is value in such an approach for limiting potential for misuse of hardware that is noncompliant in connection status, sold secondhand, stolen, and so on.

## Discussion

Across the IGSC respondents, some major benefits of membership became clear. First, IGSC members get access to the RPD, reducing the necessity for the creation of an internal SOC database. IGSC members can also take advantage of a consortium-wide reporting mechanism for suspicious orders, allowing providers to coordinate and monitor the landscape of biosecurity threats in the synthetic DNA field. Following IGSC guidelines standardizes (to the degree that it can consider country-specific nuances) screening procedures across international lines.

Finally, IGSC members participate in a robust culture of information sharing. Continued dialogue with other synthetic DNA providers and stakeholders allows providers to consistently tweak their procedures and reduces the burden on individual providers of constantly monitoring shifting synthetic DNA policies. Information-sharing seems to be of special importance to the new providers interviewed, which can gain from interorganizational dialogue as they create their screening policies.

It is noteworthy that members of the IGSC were overrepresented in our respondent pool, as might be expected, because these companies are more likely to both be stewards in the space and to utilize industry best practices (and, therefore, more forthcoming with requests for interview). This group is also most likely to be actively participating collaboratively in improving sequence screening procedures, giving insight into best practices, common roadblocks, and necessary precautions, about which less-involved companies may not have been able to speak.

Thus, although the conclusions we can draw about practices in use are primarily representative of IGSC members, the reflections on screening capabilities, provider needs, and challenges in the space are broadly applicable. It is also important to acknowledge that the respondents are likely a concentration of the most responsible entities in the space, as companies who self-evaluate that their procedures may draw scrutiny would have been less interested in responding to interview requests. It is not unreasonable to conclude that the perspectives in this article thus reflect the very best practices in place, and that the overall state of biosecurity practices in the field is likely less rigorous than represented in this study; perhaps much less rigorous.

All three non-IGSC respondents were nontraditional DNA providers (i.e., are not doing de novo gene synthesis) of the opinion that IGSC protocols were generally not applicable to their specific niche in the field, and as such did not see a need to become a member of the consortium. Two of these providers, which were focused on oligonucleotide synthesis and high-throughput replication of premade sequences, do not follow any regulations at all, stating that none exist that are relevant to them. These nontraditional providers may not be as easy for bad actors to exploit to receive SOCs, as most of them can control which presynthesized sequences enter their systems, but it is worth highlighting that entities exist that operate relatively outside of current synthesis industry norms.

The variability between IGSC organizations in terms of their lower baseline in screening is representative of a larger debate in the field regarding how to accurately screen sequences while reducing false positives and cost. Decreasing the minimum length screened allows providers to better capture short SOC. However, the shorter the sequence evaluated, the more likely it is for a sequence to match a bevy of genes across a broad range of organisms, ratcheting up the rate of false positives, and consequently time cost.

Those providers who decided to preemptively dip below the IGSC-recommended 200 nt bound for screening may simply have been anticipating the new DHHS guidelines, but they also are demonstrating a willingness to increase the potential cost of screening in favor of a higher level of security. Those that currently remain at 200 nt, in contrast, are likely guided in their decision by the high false-positive rate and cost of review. Considering that past commentary expressed concern about providers bearing the burden of the high costs of screening,^23,24^ the general attitude of acceptance toward cost is a clear success of industry norms.

At the Federal level, there is limited guidance on how best to screen short synthetic oligonucleotides. In April 2022, DHHS released a draft *Screening Framework Guidance for Providers and Users of Synthetic Oligonucleotides* that recommended that providers screen orders of oligonucleotides of at least one micromole quantity of length of 20 nt or more, and flag as an SOC any combination of oligonucleotides that is best aligned to an SOC of at least 50 nt.^9^ This tactic reflects the community's concern toward the danger of unscreened oligonucleotides being used to construct dangerous sequences piecemeal.

However, this language was removed from the final Guidance due to feedback from the community, concerned about the scientific and practical feasibility of screening such short lengths.^25^ The variability of approaches to oligonucleotide screening described by interviewees evinces that guidance for providers producing or screening oligonucleotides is currently insufficient. It is critical that upcoming guidance and/or policy provides clear and thorough approaches for oligonucleotide screening as a safeguard against the reconstruction of SOCs from short oligonucleotide sequences, which should be developed through consultation with synthetic DNA providers.

When asked about methods to mitigate the cost of implementing screening procedures, a contradiction arose over whether internal or external (contracted) screening services are more cost-effective. It is possible that the strategy that is better for cost-reduction for an individual provider may have to do with the quantity of orders they receive in a given period of time, but this was not conclusive from our interviews. Further research should be conducted on the benefits and drawbacks of building internal SOC databases versus contracting sequence screening out to a service provider.

Although BLAST provides a free tool to assist in the screening procedure, it has both technical and applied limitations. BLAST “best match” approaches for pathogens can suffer from taxonomic categorization inaccuracies, which are particularly acute for highly studied taxa.^26^ In addition, this tool requires an SOC database for comparison, the construction cost of which can be high for new or small providers; so, BLAST is not an end-all solution for the cost of screening. Similar cost sentiments can be applied to contracting out screening to other companies, although there are clear benefits to this strategy for nascent providers in overcoming initial technical hurdles to providing synthesis services.

In addition to services like those of Aclid and Battelle that have historically been contracted for DNA screening, new SOC databases and screening platforms are also in the process of being developed, such as that of the Secure DNA Project.^27^ However, wide adoption of specific SOC databases presents an information hazard, making breaches of such centralized databases more broadly dangerous. Careful consideration in guidance and regulation is needed concerning the security measures that would be requisite of more multilateral screening methodologies.

Although two synthetic DNA providers and stakeholders conduct randomized or regular red-teaming and running of test sets as a form of M&E, many providers reported that their standard review of flagged sequences during the normal ordering process is sufficient to monitor the effectiveness of their screening procedures. This sort of human review can catch false positives, which will occur at higher rates with upcoming lower length bounds. However, any false negatives a system produces will be missed by this sort of review. Thus, regular red-teaming and test set challenges represent a more comprehensive strategy for M&E.

Currently, the IGSC mandates that a test set be run by member organizations upon onboarding, but further testing is not required past this point.^28^ Expansion of IGSC policy to dictate more stringent M&E would be influential for M&E uptake across the field. The M&E procedures for synthetic DNA providers may change entirely as the U.S. government creates guidelines for stress-testing screening systems pursuant to *Executive Order 14110*, and it would be sensible for adoption of regular red-teaming and/or test set challenges to be part of resultant guidance or regulation.^11^

The greatest vulnerability identified by this study is the surprisingly small number of providers with formalized procedures for law enforcement reporting in the case of a potentially dangerous order. It is true that many respondents have never had to report an order to law enforcement before, but this obviously does not preclude such an event from occurring in the future. In addition, although the lack of formalized laws governing gene synthesis means that there is no mandated method to interface with law enforcement, both the 2010 and 2023 HHS Guidance call on gene synthesis companies to report suspect activity to an FBI Weapons of Mass Destruction Coordinator.^10,15^

Thus, it is critical that U.S.-based providers at the very least establish a relationship with their local FBI Weapons of Mass Destruction Coordinator. International providers will need to identify other points of contact in their country's national security apparatus, which is an issue that the IGSC could assist with in the near term, and that any governance of the future should address. In the long term, governments must establish clear lines of communication with synthetic DNA providers such that there is no ambiguity in which cases must be reported to law enforcement. The IGSC could also reevaluate the utility of and messaging around its internal reporting mechanism, as this tool is only useful if entities are contributing to it.

Many providers approached the issue of screening international purchases by universalizing their sequence screening procedures and SOC databases for all markets they service. Even when sequence screening procedures are standardized between domestic and international purchases, shipping synthetic genetic material across country lines requires added scrutiny. A large proportion of sequences that are flagged by screening tools are not necessarily dangerous, but require additional licensing to be exported by a provider or imported by a purchaser.

Some providers report that a purchaser receiving an import/export license from a Federal agency (1) increases the confidence of the provider that the purchaser is legitimate and (2) removes some of the burden of information-gathering and customer screening by the provider. For domestic providers, the U.S. Bureau of Industry and Security assists with the licensing process, serving as an example of how governments can support the safe import and export of genetic material.

International providers interviewed did not specifically identify analogous government organizations in their home country, so it is unclear whether these governments need to take an increased role in the transfer of synthetic DNA, or if the infrastructure exists but is not known to those providers. In the private sphere, international shipping companies such as FedEx and in-house trade compliance teams can also assist with the licensing procedure. In-country distributors can similarly be useful for navigating licensing laws, with the added benefit of assisting in customer screening with third-party purchasers in foreign countries.

The consistent hesitance of providers to whitelist purchasers of SOCs or publicize their screening procedures represents the coalescing of a broader “zero-trust model” in the DNA synthesis field. Under such a model, synthetic gene providers treat every purchase as potentially dangerous until proven otherwise by rigorous screening, and write and implement procedures related to biosecurity (including practices around transparency and data storage) with an abundance of caution.

However, there is some debate when it comes to transparency in screening workflows. The few providers who do completely publicize their procedures argue that screening processes should be so airtight that bad actors should not be able to get around them regardless of how publicly available they are. Whether this inviolability of screening procedures is possible for the entirety of the DNA synthesis field is unclear. For now, the majority of providers interviewed prefer to err on the side of caution, working under this model of zero-trust and low transparency.

Outside of transparency, providers deciding to lower their screening bounds below the IGSC's 200 nt guidance and maintaining data on customers and purchases longer than the IGSC-recommended 8 years are both examples where a hypervigilant zero-trust mindset can manifest. Privatized DNA synthesis is also a relatively new field, with no regulation and high degrees of uncertainty in regard to biosecurity. It is possible that with further biosecurity frameworks, the field will feel more secure in beginning to publish their procedures.

The advent of benchtop synthesis devices opens up a new frontier in synthetic genomics that calls for innovative ways to rethink sequence screening. For example, there is currently an ongoing debate in the field on whether DNA synthesizers should employ a cloud-based “phone-home” model of screening or whether screening should be conducted entirely locally, with SOC screening algorithms built into the device itself. Although the benchtop synthesis providers surveyed in this study largely employed the phone-home method, they should not be taken as a representation of the entire benchtop synthesis field, as it was not the goal of this analysis to address that issue.

Separate more exhaustive reporting of benchtop synthesis providers has been conducted and should be consulted for a more comprehensive understanding of the benchtop synthesis field specifically.^22^ Moreover, although all three of the benchtop synthesizer companies interviewed were IGSC members, the IGSC does not explicitly address benchtop synthesis devices in their Protocol, a gap that should be addressed in the next update.^7^ Outside of benchtop device companies specifically, regulatory frameworks (both within and outside of the IGSC) must be expanded to cover the nontraditional synthetic DNA providers discussed in this report that currently are operating without guidance.

As mentioned earlier, the U.S. DHHS recently finalized their updated Screening Framework Guidance for Providers and Users of Synthetic Nucleic Acids. Major changes include the expansion of the definition of SOCs to any sequence that could contribute to pathogenicity or toxicity, even if it is not related to a U.S. select agent or toxin. Moving away from pathogen threat-list-based SOC strategies to those that focus more on the function of individual sequences is gaining momentum in the field.^29,30^

However, painting with broad strokes in regulation can have deleterious consequences for research and innovation,^31,32^ and should be extremely carefully considered during DHHS' work to develop more detailed DNA synthesis screening guidance and/or regulation. The new Guidance also reduces the recommended lower bound for screening to 50 nt and calls for the screening of all single- and double-stranded DNA and RNA, although it is unclear how technically feasible this type of short-sequence screening will be in practice, while avoiding high false-positive rates.

As we found in our interviews, synthesis providers generally are already doing this work, and should be consulted directly about their technical approaches when building new guidance and/or regulation. Relevant to benchtop synthesis companies, the Guidance introduces more robust guidelines for screening of sequences and users of benchtop synthesis devices.^10^ In regard to some of the topics described by this article, it is very likely that how SOC databases are compiled, how benchtop synthesis providers approach screening, and the average lower bound utilized by providers for sequence screening will change in response to this new Guidance.

But although these changes are significant, they are not binding. Thus, although it is likely that most providers that follow DHHS Guidance will update their screening protocols accordingly, it is not a guarantee. Research support by the biosecurity community, especially as providers attempt to navigate what sequences to designate as having the potential to confer pathogenicity or toxicity, will help facilitate the smooth implementation of the new guidance for the next 3 years.

## Conclusion

DNA synthesis is a rapidly growing field, in which advances threaten to outpace regulatory policy. To safeguard the field against emergent risks, it is imperative that policymakers, companies, and end users participate in deliberations on how to best approach security infrastructure. By detailing the practices of DNA synthesis providers through interviews with these providers and stakeholders, we have provided an important resource for such ongoing deliberations on the if, when, and how of guidance and governance of synthetic DNA technologies.

It is our hope that the earlier-presented description of the current state of play and policy vulnerabilities in synthetic sequence screening will play a major role in bringing policy up to speed with innovation, and in doing so, reduce the biosecurity risks therein.

## Supplementary Material

Supplementary Data
