# Supplementary material for: Screening State of Play: The Biosecurity Practices of Synthetic DNA Providers
Source: Appl Biosaf. 2024 Jun 20;29(2):85–95. doi: 10.1089/apb.2023.0027 (PMC11319849; doi:10.1089/apb.2023.0027)
Supplement: Supplementary Data [file apb.2023.0027_suppl_data.pdf]

Supplement. Interview Questions Used in this Study

General questions about screening processes

1. What is your role in your organization, and to what degree are you personally involved with sequence screening procedures?
2. What was the process by which your screening procedures were originally created?
3. Have you revised your procedures since you first created them, and if so, how did you conduct the revision process?
4. Do you work with any entities outside your company to develop or implement DNA screening procedures? If so, who?
5. Were your screening procedures designed for compliance with any laws or international frameworks? If so, which?
6. What data do you collect on purchasers and orders, and how long do you maintain the data for?
7. Does your organization have a monitoring and evaluation framework in place to regularly validate that screens are being conducted properly?
8. Do you find your screening procedures to be costly? What do you do to mitigate the cost of screening (i.e., is the cost passed onto the consumer)?
9. Is your screening procedure for the purchase of oligonucleotides different from the general DNA screening procedure? If so, how?
10. What is your lower bound (in base pairs) for screening? Has this number changed over time?

Security in screening

11. What technology do you use (both hardware and software platforms) to conduct screenings? What databases do you use to check DNA sequences?
12. To what degree are your screening procedures publicly available? What do you do to mitigate the security risks associated with transparency in the screening process?
13. How do you designate DNA sequences of concern?
14. What procedures do you follow for purchaser screening? Do these change if you know or suspect that the purchaser intends to resell the sequence to a third party?
15. Does your company have a predefined list of legitimate purchasers of sequences of concern? If so, how is it developed and maintained?
16. If a potential purchase is flagged for containing a sequence of concern, how do you proceed? How do you decide whether to move forward with the order, and who makes that decision?
17. What percentage of orders get flagged as suspicious? What percentage of flagged orders get denied?
18. Are there any other "red flags" outside of the purchaser and the sequence that can cause an order to be considered suspicious?
19. What steps are taken if a potential purchase is determined to be too dangerous to continue?
20. Do you have a reporting mechanism for law enforcement entities in the event of a suspicious order? If so, what is it, and who do you report to?
21. Is your screening procedure different for international purchases? If so, how? Do you collaborate with any in-country organizations when carrying out screening of international purchases?
